# No *Plasmodium falciparum* Chloroquine Resistance Transporter and Artemisinin Resistance Mutations, Haiti 

**DOI:** 10.3201/eid2411.180738

**Published:** 2018-11

**Authors:** Jeanne P. Vincent, Kanako Komaki-Yasuda, Alexandre V. Existe, Jacques Boncy, Shigeyuki Kano

**Affiliations:** National Center for Global Health and Medicine, Tokyo, Japan (J.P. Vincent, K. Komaki-Yasuda, S. Kano);; University of Tsukuba, Tsukuba, Japan (J.P. Vincent, S. Kano);; Laboratoire National de Santé Publique, Port-au-Prince, Haiti (A.V. Existe, J. Boncy)

**Keywords:** Plasmodium falciparum, parasites, chloroquine, artemisinin, drug resistance, malaria, malaria elimination, P. falciparum chloroquine resistance transporter gene, pfcrt, artemisinin resistance gene, kelch 13, k13, mutations, Haiti

## Abstract

We obtained 78 human blood samples from areas in Haiti with high transmission of malaria and found no drug resistance–associated mutations in *Plasmodium falciparum* chloroquine resistance transporter and Kelch 13 genes. We recommend maintaining chloroquine as the first-line drug for malaria in Haiti. Artemisinin-based therapy can be used as alternative therapy.

Haiti is a unique country in the Americas because malaria is caused there mainly by *Plasmodium falciparum*. Despite chloroquine being used for treatment of malaria since 1955, *P. falciparum* is generally still susceptible to this drug (*1*). Thus, chloroquine, plus a single dose of the gametocytocidal drug primaquine, is still the first-line treatment for uncomplicated malaria in Haiti, as indicated by the ministry of health. This regimen began to be challenged 9 years ago after a study reported chloroquine-resistant haplotypes in Haiti (*2*). Since that time, other studies have reported no or few chloroquinine-resistance haplotypes (*3**–**6*), but an in vivo study reported a decrease in susceptibility to this drug (*7*).

Artemisinin, has been used only sporadically in Haiti, but it was recently implemented by health authorities to be the second-line antimalarial drug. We evaluated 2 drug resistance markers, the *P. falciparum* chloroquine resistance transporter (*pfcrt*) gene and the artemisinin resistance gene Kelch 13 (*k13*), in malaria parasites in Haiti to determine prevalences and provide information and recommendations for clinical practice to support malaria elimination efforts.

We conducted an epidemiologic survey during the summer of 2017. The study protocol was reviewed and approved by the Ethics Committee of the National Center for Global Health and Medicine (reference no. NCGM-G-002260–00) in Japan and the National Bioethics Committee (reference no. 1617–48) in Haiti.

We recruited febrile patients at 3 public hospitals in 3 departments in southern Haiti. We tested these patients by using a rapid diagnostic test (SD Bioline Malaria Ag Pf/Pan; Standard Diagnostics, Inc., Suwon, South Korea) at the point of care. These patients were a subsample of 556 patients from which we selected 144 patients with blood samples positive for *P. falciparum* DNA by the loop-mediated isothermal amplification method (Loopamp MALARIA Pan/Pf Detection Kit; Eiken Chemical Co., Tokyo, Japan). These 144 patients were potentially eligible for genotyping analysis. 

We confirmed 80 positive samples from these patients by using a nested PCR specific for the 18S rRNA gene for analysis of *pfcrt* and *k13* genes. Conditions for this nested PCR were as reported (*8*). We performed the second PCR with only *P. falciparum*–specific primers. We amplified the *k13* gene by using a modified method of Ménard et al. (*9*) and newly designed primers specific for the *pfcrt* gene (Table). For *pfcrt* or *k13* genes, secondary PCR products were sequenced directly.

**Table:** Primers used for nested PCRs to detect *Plasmodium falciparum* chloroquine resistance transporter and artemisin gene resistance mutations, Haiti*

Target	Primer sequences, 5′→3′	Primer annealing positions
*pfcrt*, primary PCR	F: ATGGCTCACGTTTAGGTGGAGGT	92–114
	R: CGGATGTTACAAAACTATAGTTACCA	258–283
*pfcrt*, secondary PCR	F: GTCTTGGTAAATGTGCTCATGTGT	119–142
	R: CTATAGTTACCAATTTTGTTTAAAGTTCT	241–269
*k13*, primary PCR	F: GAAGCCTTGTTGAAAGAAGCA	1276–1296
	R: CCAAGCTGCCATTCATTTGT	2107–2126
*k13*, secondary PCR	F: GCCTTGTTGAAAGAAGCAGAA	1279–1299
	R: GTGGCAGCTCCAAAATTCAT	2011–2030

*Secondary PCR products were directly sequenced by using the BigDye Terminator version 3.1 Cycle Sequencing Kit and analyzed with a 3130xl Genetic Analyzer (both from Thermo Fisher Scientific Inc., Waltham, MA, USA). F, forward; *k13*, Kelch 13; *pfcrt*, *P. falciparum* chloroquine resistance transporter; R, reverse.

We analyzed samples from 78 patients for *k13* and samples from all 80 patients for *pfcrt*. The 80 patients had a mean age of 26.97 years (range 1–70 years): 13 were from Grand’Anse Department, 24 from Nippes Department, and 43 from Sud Department. Of these samples, 71 were also positive for the Pf-specific HRP2 band of the rapid diagnostic test but only 52 for the *Plasmodium*-universal LDH band. Microscopy results identified only 40 of these patients as being positive for malaria.

All samples analyzed had the wild-type amino acid sequence CVMNK at positions 72–76 of *pfcrt*. Resistant haplotypes of *pfcrt* were first reported in Haiti in 5 of 79 analyzed samples from Artibonite Department (*2*). Others studies have reported chloroquine-resistant haplotypes in 2 travelers returning from Haiti (*3*), 2/901 persons with possible mixed infections (chloroquine resistant and chloroquine sensitive) (*4*), and 1/108 cases analyzed in which microsatellite genotyping showed that the chloroquine-resistant haplotype detected was distinct from those of parasites circulating in Haiti (*5*). Analysis of parasite population structure in 2 of these studies (*4**,**5*) could not eliminate the possibility that these cases might be exogenous infections. In addition, Elbadry et al. did not report any chloroquine-resistant haplotypes in Haiti (*6*).

None of the 78 samples we tested had any resistance-associated polymorphisms in *k13*. Five (6.41%) samples had a synonymous mutation at nt position 1359 (bp position T1359A, codon position G453). This mutation was previously reported in only 1/82 samples in a study in Haiti (*10*). These findings are not an indication of artemisinin resistance because artemisinin-based combination therapy is rarely used in Haiti. However, these results are useful for following the evolution of resistance to this drug in Haiti.

In this study, we analyzed patients from areas of Haiti that have high rates of malaria transmission and found no drug resistance–associated mutations for the *pfcrt* and *k13* genes. Despite the limitation of a small sample size and consideration of findings of previous studies and our recent findings, we can assert that drug-resistant haplotypes are not currently circulating in Haiti.

Affordable and widely available, chloroquine is still the treatment of choice for uncomplicated *Plasmodium* spp. malaria in Haiti. Artemisinin-based combination therapy can be used as an alternative treatment for persons who cannot be given chloroquine. Although posttreatment follow-up visits with blood testing of malaria patients can be challenging in Haiti, healthcare professionals should strive to implement these goals. Implementation would enable continuous in vivo monitoring of drug susceptibility of parasites and provide real-time data to public health authorities to formulate evidence-based policy.
